# The effectiveness of Kayaking exercises as compared to general mobility exercises in reducing axial rigidity and improve bed mobility in early to mid stage of Parkinson’s disease

**DOI:** 10.12669/pjms.305.5231

**Published:** 2014

**Authors:** Faiza Shujaat, Nabila Soomro, Muhammad Khan

**Affiliations:** 1 Faiza Shujaat, MSPT, Institute of Physical Medicine & Rehabilitation, Dow University of Health Sciences Karachi Pakistan.; 2 Dr. Nabila Soomro, FCPS, Institute of Physical Medicine & Rehabilitation, Dow University of Health Sciences Karachi Pakistan.; 3 Muhammad Khan, MSc.PT, Institute of Physical Medicine & Rehabilitation, Dow University of Health Sciences Karachi Pakistan.

**Keywords:** Parkinson’s disease, rehabilitation, axial rigidity, kayaking exercises, physiotherapy

## Abstract

***Objective:*** To determine the effectiveness of kayaking exercises in the management of axial rigidity, improve bed mobility by improving trunk rotation in Parkinson’s patients.

***Methods:*** Experimental randomized controlled trail conducted at Physiotherapy department of IPM&R, DUHS and neurology Outpatient Department of Civil Hospital Karachi. Sample size of 48 was calculated with the use of openEpi. After baseline assessment 24 participants were assigned to each Kayaking exercise and general mobility exercise groups. Both groups received treatment for 75 minutes, 6 days a week for 4 weeks. Pre and post treatment measurements were determined by goniometer that assessed the cervical and thoracolumbar rotations whereas bed mobility was assessed by Modified Parkinson’s Activity Scale (MPAS).

***Results: ***In Kayaking group mean cervical spine left rotation increased from 32.95+ 9.66 to 47.25 + 10.58, right side cervical spine rotation increased from 34.00 + 10.32 to 47.58 + 11.96, left side thoracolumbar rotation increased from 23.67 + 4.70 to 28.16 + 3.44, right side thoracolumbar rotation increased from 20.79 + 5.34 to 26.45 + 4.62. In control group mean cervical spine left rotation increased from 34.66+ 9.26 to 43.08 + 8.70, right side cervical spine rotation increased from 35.37 + 9.77 to 43.83 + 9.59 , left side thoracolumbar rotation increased from 23.70 + 4.77 to 26.87 + 3.73, right side thoracicolumbar rotation increased from 21.16 + 5.29 to 24.95 + 4.53 (P value <0.001).. Bed mobility on MPAS scale also showed significant improvements (P value <0.001).

***Conclusion:*** Both Kayaking and general exercises resulted in significant improvements after 4 weeks of treatment. However, Kayaking exercises were slightly more beneficial than general exercises.

## INTRODUCTION

Parkinson’s disease (PD) is the 2^nd^ most widespread neurodegenerative progressive disorder of elderly.^1^ According to Dorsey Ray, worldwide number of Parkinson’s disease will double in next 25 years especially in under developed countries including Pakistan.^2^ A study conducted at Agha Khan University hospital of Karachi identified 81 Parkinson’s patients out of 101 patients in 11years of time span.^3^ Idiopathic Parkinson’s disease begins most often between the decades of 40’s and 60’s.^4^ PD is characterized by a series of motor and non-motor symptoms that affect a person’s functional ability to a great deal these motor and non-motor symptoms give rise to secondary impairments like stooped posture (camptocormia), gait impairments and difficulty in mobility.^5^ Apart from medication other treatment options available for PD are physical therapy, surgical intervention and deep brain stimulation (DBS) but DBS is not a cost effective treatment, also its efficacy and safety are studied in short term studies.^6^

Physical therapy is usually prescribed along with medical treatment. Hirsch *et al*,^7^ & Dibble *et al,*^8^ have suggested that strengthening and balancing exercises would be helpful in managing the stride length and balance in PD. Although it would not influence the disease process but can help in maintaining and improving activities of daily of living (ADLs) and quality of life (QOL). Physical therapy targets on six specific core areas in PD that includes posture, transfer, dexterity, balance, falls, gait problems and endurance for physical activities.^9^ Schenkman and Butler suggested improvement in axial rigidity and its related complications in people who actively participated in Physiotherapy.^10^ Horak suggested kayaking exercises for improving axial rigidity. Axial rigidity is controlled by different neuronal circuits that are different from the one that control appendicular rigidity. Some literature suggests beneficial result of Levodopa therapy and deep brain stimulation in improving axial rigidity.^10^^,^^11^ There are also evidences that report adverse effects of long term use of Levodopa therapy.^12^ Whereas literature about physiotherapy has reported beneficial results in improving gait, fall and balance problems.^9^^,^^11^

At present many studies have suggested optimistic reports of physical therapy programs. These programs helped in improving the ADL and QOL in PD patients. Present study is a quasi-experimental trial in Parkinson diseased patients which is based on suggestive study of King and Horak. They referred kayaking exercises to be helpful in improving axial rigidity.^11^ This study was planned to demonstrate the effectiveness of kayaking exercises for the same purpose.

Kayaking exercises consisting of big, flowing, multi segmental movements of whole body and involve rotatory movements of trunk.

## METHODS

This study was a single blinded, quasi experimental study conducted in Institute of Physical Medicine & Rehabilitation (IPM&R). Patients were selected from OPD of neurology Civil Hospital Karachi. Both males and females’ age 35-65 years who were diagnosed with idiopathic Parkinson’s disease stage 1-3 on Hoehn & Yahr scale and had no associated medical condition were included. Patients with dementia, Alzheimer disease, stroke and other movement disorders like parkinsonian plus syndromes were excluded. The study was conducted between May 2011 and December 2011 after approval from institutional review board (IRB) of Dow University of Health Sciences. Sample size of 48 individuals, 24 each group, was selected, with α = 10% and power = 90%.

Forty eight subjects’ fulfilled the inclusion criteria and were included to participate in the study. After taken written consent purposive sampling technique was used to assign subjects into two groups. Group ‘A’ was experimental group and included kayaking exercises consisting of big, flowing, multi segmental movements of whole body and involves rotatory movements of trunk. Group ‘B’ included strengthening exercises of the upper limbs, lower limbs and core stabilizers. Both groups started their sessions with breathing exercises and warm ups which were proceeded by stretching exercises of neck, arms, legs and trunk. All the exercises were demonstrated to the patients by the physical therapist and were performed under the supervision of the physical therapist. Therapy duration was of four weeks, numbers of sessions were 24 and each session was of 75 minutes, 6 days per week. Pre and post treatment evaluation of bed mobility and axial rigidity was measured using the modified Parkinson’s activity scale (MPAS). MPAS is a comprehensive practical test for gait and transfers (including rolling over in bed). The test is a valid and reliable instrument for patients with PD and gives relevant information for the diagnostic and therapeutic processes. It is scored from 0 to 4. Table-I. Range of motion of cervical spine and thoracolumbar spine (right and left rotation) was measured with goniometer.

**Table-I:** Activity score Modified Parkinson’s Activity scale

***Score***	***Activity level***
0	Dependent,
1	Task is 3 times difficult
2	Task is 2 times difficult
3	Task is 1 time difficult
4	Normal

SPSS version 20 was used for data analysis. Wilcoxon signed rank test was used to reveal the effects of treatments within the groups. Mann Whitney U test was used to compare group A and group B for their pre and post treatment effects with level of significance P-value less than 0.05 considered significant.

## RESULTS

A total of 48 patients participated in the study and with no drop out. The inclusion criterion was 35 to 65 years with early to mid-stage of disease severity on Hoehn & Yahr scale but, in this study patients were found to lie in age group of 40- 70 years due to difficulty to complete sampling size in the age ranges from 35 to 65. In experimental group mean (SD) duration of disease (years) among participants was found to be 56.50 (9.76) whereas in control group it was 56.33(10.35). On Hoehn and Yahr stage scale in experimental group there were 54.17% whilst in control group there were 70.08% participants in stage 1.5 respectively; in experimental group 37.50% participants and in control group 20.83% participants were in stage 2. In stage 2.5 and 3 there were 4.17% participants in each group.

There were 87.50% (42 out of 48) male cases and 12.50% (6 out of 48) were female. Hence there was a significant difference in male and female proportion in both groups.

Intra group analysis which showed significant difference in the degree of rotation and MPAS IIIA & B in both treatment groups (p value < 0.001). However, kayaking group ranges and MPAS scores were slightly better than control group (Tables II and III).

**Table-II:** Pre and Post treatment comparison for rotation of cervical and thoracolumbar spine in kayaking and control groups

***Treatment Groups***	***Rotation***	***Mean (SD)***		***p- values***
		***Pre Treatment***	***Post treatment***	
*Kayaking Group*	CS Lt	32.95 + 9.66	47.25 + 10.58	<0.001^*^
	CS Rt	34.00 + 10.32	47.58 + 11.96	<0.001^*^
	TLS Lt	23.67 + 4.70	28.16+ 3.44	<0.001^*^
	TLS Rt	20.79+ 5.34	26.45 + 4.62	<0.001^*^
*Control Group*	CS Lt	34.66 + 9.26	43.08 + 8.70	<0.001^*^
	CS Rt	35.37 + 9.77	43.83 + 9.59	<0.001^*^
	TLS Lt	23.70 + 4.77	26.87 + 3.73	<0.001^*^
	TLS Rt	21.16 + 5.29	24.95 + 4.53	<0.001^*^

**Table-III:** Pre and Post treatment comparison for MPAS III bed mobility in kayaking and control groups

***Treatment*** ***Groups***	***MPAS – III*** ***Bed Mobility***	***Mean (SD)***		***p-value***
		***Pre treatment***	***Post treatment***	
*Kayaking Group*	III A WOC	11.20 + 3.42	14.08 + 3.10	<0.001^*^
	III B WC	10.83 + 4.31	13.45 + 4.03	< 0.001^*^
*Control Group*	III A WOC	11.12 + 3.34	13.75 + 3.02	< 0.001^*^
	III B WC	10.83 + 4.33	13.04 + 3.98	< 0.001^*^

Mann Whitney U test was performed to test the significance of changes between the two groups and found no significant difference in the degree of rotation and scores of MPAS III A and B (p value MPAS IIIA = 0.819 MPAS IIIB= 0.983) between the two groups before and after treatment (tables 4 and 5; showing P – values < 0.001). 

## DISCUSSION

This study examined the short term benefits of two modes of exercises in improving axial rigidity that shows little response by pharmacological treatment.^11^^,^^13^ The results showed that both mode of exercise have significantly improved cervical spine and thoracolumbar spine left and right side rotation range of motion in both treatment groups measured with goniometer (p-value < 0.001). Bed mobility with cover and without cover also significantly improved in both treatment groups (P-value < 0.001) measured with modified Parkinson’s activity scale (MPAS).

To the best of our knowledge this was the first study looked at the effectiveness of kayaking exercises over general mobility exercises in improving the axial rigidity and bed mobility in Parkinson’s patients in Pakistan.

According to Hoehn and Yahr scale axial rigidity manifests its symptoms in the 1.5 stage of the disease (stage 1.5 –stage 3 of Hoehn and Yahr scale represents early to mid-stage of the disease). Work of Schenkman also support that spinal range of motions are affected in early stage of the disease and showing better results if involved in exercises in this stage.^14^^,^^15^ On the basis of previous researches of Schenkman *et al.,*^15^ this study included patients from 1.5 to 3 Hoehn and Yahr stage of PD because these stages are characterized by axial involvement without loss of balance and showed significant improvements in both groups. The results of this study also found kayaking and other general mobility exercises are successful in early stages of the disease.

In Axial rigidity spinal movements mostly impaired are extension and rotation and patients tends to adapt a flexed posture that reduce their functional movements and impairs sleep.^14^ Therefore this study was focused on the rotations of cervical and thoracolumbar spine. Serrao *et al*.^16^ and Roberto *et al.*^17^ studied flexion, extension and lateral flexion movements and reported improvement in extension and lateral flexion but no improvement was reported in rotation. King and Horak^11^ devised a sensory motor agility program for Parkinson’s patients and suggested kayaking to be helpful in improving axial rigidity. Thus the effects of kayaking exercises were studied in improving axial rigidity and bed mobility along with general mobility exercises which were designed by Canadian society of Parkinson’s disease. As far as author’s knowledge is concerned, there is little data published on the effects of kayaking exercises on axial rigidity in improving bed mobility. However many patients do exercise kayaking as leisure activity in western countries^18^ but not in Pakistan. According to the results of our study trunk flexibility can be improved by kayaking exercises which supports the findings of Schenkman and his colleagues.^10^^,^^15^^,^^19^

Recently, Schenkman and colleagues^20^ found that flexibility, balance and functional exercises were more beneficial in improving overall functional ability. Our study did not include the overall functional ability but just focused on the bed mobility which is one of the functional problems faced by PD patients and showed positive results. The improvement in rotations and MPAS scores in current study are in concurrence to the findings of^15^ that early rehabilitation in PD patients benefits the PD sufferers. Participants of the control group also showed equally good response to the general exercises as kayaking group participants, which might be due to the reason that majority of these participants, before entering the research were actively involved in general Parkinson’s rehabilitation program. Whereas kayaking group participants were all new to any post Parkinson’s rehab program. However, participants of kayaking exercises showed better improvements in ranges and on MPAS as compared to the general mobility exercises. The difference in results between Kayaking group and control group might be due to the reason that kayaking movements include alternate limb girdle movement that diminishes the reciprocal movements and facilitates rhythmic movements which are lost or minimized due to basal ganglia defect.^10^ Although, in this study, no subjective outcome measure was used, but people in kayaking group reported functional improvements in terms of various movements e.g. 2-3 patients reported less resistance while performing their prayers like sajda and rukoo, or in overhead activities. Since axial rigidity not only affects trunk rotations but also have great impact on girdle limb association.^21^

In this study, degree of rotation at cervical spine and thoracolumbar spine were measured and modified Parkinson’s activity scales were selected as outcome measures. Although studies related to spinal range of motion used functional axial rotation test (FAR) or twister, ^22^ but none of the method was feasible in our scenario. Therefore universal goniometer was used for measuring cervical and thoracolumbar degree of rotation and Modified Parkinson’s activity scale was used to assess bed mobility. MPAS was used as it proved to be a better instrument as it measures the details of every movement. It showed good concurrent validity (0.64) and (0.79) with UPDRS III and VAS global functioning respectively.^22^

There were 24 patients in each group with 3 females and 21 males which is similar to the fact that PD is more prevalent in males than females.^3^ About 62.12% of participants’ were in Hoehn and Yahr stage 1.5 supporting the view that axial involvement exist in early stage of the disease.^3^ All of them belong to the age group from 40 to 70 years (37.50%); of them majority of patients lie in the age group of 50 to 70 years which is similar to the findings of other studies that PD is more prevalent in old age.^3^ It is also evident in young age group as in this study approximately 25% participants belonged to young age group. Although, the inclusion criteria of this study limit the age from 35 to 65 years but, when participants were assessed for the study not a single patient was found in the age of 35. However, during screening there were patients who belong to this age group but they did not have axial rigidity or impaired bed mobility. In the same manner patients with age 70 were enrolled in this study as their severity of disease was lying in mild to moderate range at Hoehn and Yahr scale with impaired bed mobility. In current study, duration of disease among the participants was found to be 1 to 2 years and 3 to 4 years also supporting the Hoehn and Yahr stage which suggests axial involvement begins in early to mid-early stage of disease.^23^

All patients in this study were on medications as prescribed by the doctor, majority of them were on combination of drugs. Different people were on different combinations and we were unable to homogenize the medication among all as it may have negative impact on their health status. However, there were patients in both groups who were not taking medicines as their doctor did not prescribe any and told them that they were not in need of medicine right now, but they were having difficulty in overhead activities and rolling. They reported better improvement to the treatment supporting the view that exercises helps in improving dopamine functioning.^6^

***Limitations of the study: ***The assessor of the study was not blind and the participants in control group were actively involved in a supervised exercise group before entering the research which could have impact on results. All participants were on different medicines and it was a short duration study without long term follow up.

## CONCLUSION

In conclusion, both Kayaking and general exercises resulted in significant improvements in cervical spine and thoracolumbar spine left and right side rotation range of motion and bed mobility after 4 weeks of treatment. However, Kayaking exercises were slightly more clinically beneficial than general exercises. Further research is required to explore this further. 

## Authors Contribution:

**FS:** Topic selection, design, data collection and anylysis.

**NS: **Topic selection, designing the study.

**MK:** Manuscript writing, literature search.

## Notes

***Grant Support & Financial Disclosures:*** None.

## References

[1] Nutt JG, Wooten GF (2005). Clinical practice: Diagnosis and initial management of Parkinson's disease. N Engl J Med.

[2] Dorsey ER, Constantinescu R, Thompson JP, BiglanK M, Holloway RG, Kieburtz K (2007). Projected number of people with Parkinson disease in the most populous nations, 2005 through 2030. Neurology.

[3] Khealani BA, Baig SM (2006). Clinical spectrum of Parkinson’s disease from Pakistan. Singapore Med J.

[4] McPhee SJ, Papadakis MA (2009). McPhee Current medical diagnosis & treatment.

[5] Jankovic J (2008). Parkinson's disease: clinical features and diagnosis. J Neurol Neurosurg Psychiatry.

[6] Jankovic J, Aguilar LG (2008). Current approaches to the treatment of Parkinson’s disease. Neuropsychiatr Dis Treat.

[7] Dibble LE, Hale TF, Marcus RL, Droge J, Gerber JP, LaStayo PC (2006). High-intensity resistance training amplifies muscle hypertrophy and functional gains in persons with Parkinson's disease. Mov Disord.

[8] Hirsch MA, Toole T, Maitland CG, Rider RA (2003). The effects of balance training and high-intensity resistance training on persons with idiopathic Parkinson's disease. Arch Phys Med Rehabil.

[9] Keus SH, Bloem BR, HendriksEJ, Bredero-Cohen AB, Munneke M (2007). Evidence-based analysis of physical therapy in Parkinson’s disease with recommendations for practice and research. Mov Disord.

[10] Schenkman M, Butler RB (1989). A model for multisystem evaluation treatment of Individuals with Parkinson's disease. Phys Ther.

[11] King LA, Horak FB (2009). Delaying mobility disability in people with Parkinson disease using a sensorimotor agility exercise program. Phys Ther.

[12] Jankovic J, Stacy M (2007). Medical management of Levodopa-associated motor complications in patients with Parkinson’s disease. CNS Drugs.

[13] Massano J, Bhatia KP (2012). Clinical approach to Parkinson’s disease: features, diagnosis, and principles of management. Cold Spring Harb Perspect Med.

[14] Vaugoyeau M, Viallet F, Aurenty R, Assaiante C, Mesure S, Massion J (2006). Axial rotation in Parkinson's disease. J Neurol Neurosurg Psychiatry.

[15] Schenkman ML, Clark K, Xie T (2001). Spinal movement and performance of a standing reach task in participants with and without Parkinson disease. Phys Ther.

[16] Serrao M, Tassorelli C, Don R, Ranavolo A, Draicchio F, Sandrini G (2010). Four-Week Trunk-Specific Rehabilitation Treatment Improves Lateral Trunk Flexion in Parkinson’s disease. Mov Disord.

[17] Roberto C, Lydia V (2011). Axial rigidity and quality of life in patients with Parkinson’s disease: a preliminary study. Qual Life Res.

[18] Gina Shaw (2006). Janet Reno’s law of motion. NBC Universal (Saturday Night Live).

[19] Schenkman M, Cutson TM, Kuchibhatla M, Chandler J, Pieper CF, Ray L (1998). Exercise to improve spinal flexibility and function for people with Parkinson's disease: a randomized, controlled trial. J Am Geriatr Soc.

[20] Schenkman M, Hall DA, Barón AE, Schwartz RS, Mettler P, Kohrt WM (2012). Exercise for People in Early- or Mid-Stage Parkinson Disease: A 16-Month Randomized Controlled Trial. Phys Ther.

[21] Wright WG, Gurfinkel VS, Nutt J, Horak FB, Cordo PJ (2007). Axial hypertonicity in Parkinson’s disease: Direct measurements of trunk and hip torque. Exp Neurol.

[22] Keus SH, Nieuwboer A, Bloem BR, Borm GF, Munneke M (2009). Clinematric analysis of Modified Parkinson’s activity scale. Parkinsonism Relat Disord.

[23] Fahn S, Elton RL, Members of the UPDRS Development Committee, Fahn S, Calne D, Goldstein M (1987). Unified Parkinson’s disease rating scale. Recent Developments in Parkinson’s disease.

